# Supplementary material for: Complexome profiling of the Chlamydomonas psb28 mutant reveals TEF5 as an early PSII assembly factor
Source: Plant Cell. 2025 Mar 17;37(6):koaf055. doi: 10.1093/plcell/koaf055 (PMC12164590; doi:10.1093/plcell/koaf055)
Supplement: koaf055_Supplementary_Data [file koaf055_supplementary_data.zip › psb28_interactive.zip!/plots/intensity/qualityPlots/peptides/NUOB4_Cre03.g204650.t1.2.html]

### Cre03.g204650.t1.2

Trivial name: NUOB4  
SeqCoverage [median]: 31.70  
Mass: 16.17 kDa  
  
 

| Sequence | MissedCleavage | Mass [Da] | wtIntensity | muIntensity | Score |
| --- | --- | --- | --- | --- | --- |
| DHFENEFVWNYR | 0 | 1654.72 | 1973.55 | 0.00 | 78.55 |
| NPYIEAWYYR | 0 | 1373.64 | 4557.80 | 0.00 | 126.52 |
| QFTPDGK | 0 | 791.38 | 8636.32 | 6595.48 | 147.34 |
| QILGDEPAMVLPDER | 0 | 1681.83 | 8654.21 | 8075.44 | 164.11 |
| RQILGDEPAMVLPDER | 1 | 1837.94 | 13873.87 | 24269.01 | 117.00 |
|  |  |  | 37695.7 | 38939.9 |  |
